# Computed Tomography Scanner Productivity and Entry-Level Models in the Global Market

**DOI:** 10.1155/2017/1304960

**Published:** 2017-09-28

**Authors:** R. P. Santos, A. L. A. Pires, R. M. V. R. Almeida, W. C. A. Pereira

**Affiliations:** ^1^Centro Federal de Educação Tecnológica (CEFET), UnED Itaguaí, Itaguaí, RJ, Brazil; ^2^Programa de Engenharia Biomédica, Instituto Alberto Luiz Coimbra (COPPE), Universidade Federal do Rio de Janeiro (UFRJ), Rio de Janeiro, RJ, Brazil; ^3^Departamento de Engenharia Eletrônica e de Computação, Escola Politécnica, Universidade Federal do Rio de Janeiro (UFRJ), Rio de Janeiro, RJ, Brazil

## Abstract

**Objective:**

This study evaluated the productivity of computed tomography (CT) models and characterized their simplest (entry-level) models' supply in the world market.

**Methods:**

CT exam times were measured in eight health facilities in the state of Rio de Janeiro, Brazil. Exams were divided into six stages: (1) arrival of patient records to the examination room; (2) patient arrival; (3) patient positioning; (4) data input prior to exam; (5) image acquisition; and (6) patient departure. CT exam productivity was calculated by dividing the total weekly working time by the total exam time for each model. Additionally, an internet search identified full-body CT manufacturers and their offered entry-level models.

**Results:**

The time durations of 111 CT exams were obtained. Differences among average exam times were not large, and they were mainly due to stages not directly related to data acquisition or image reconstruction. The survey identified that most manufacturers offer 2- to 4-slice models for Asia, South America, and Africa, and one offers single-slice models (Asia). In the USA, two manufacturers offer models below 16-slice.

**Conclusion:**

Productivity gains are not linearly related to “slice” number. It is suggested that the use of “shareable platforms” could make CTs cheaper, increasing their availability.

## 1. Introduction

Access to modern technology reflects the level of economic development of a nation. At the same time, modern equipment is essential for medical science and clinical diagnosis. Many countries still suffer from a lack of adequate medical equipment, and organizations such as the International Atomic Energy Agency (IAEA), the Pan American Health Organization (PAHO), and the World Health Organization (WHO) support their member states to increase worldwide access to such technology [1–3].

Computed tomography (CT) is one of the most important diagnostic tools in medicine, with a wide scope for clinical use [4, 5]. In 2009, Brazil had 15.6 CT scanners (CTs) per million people, a density comparable to Canada (14.2) and New Zealand (15.8). However, Brazil's southeastern region had 21.8 CTs per million people, more than Luxembourg (19.7), and its northeastern region had 8.1 CTs per million people, fewer than Tunisia (9.3). Of all medical imaging devices in Brazil in 2009, 4.7% were CTs, ranking eighth in the number of installed devices [6–8].

In the US, the percentage of patients using CTs in emergency departments increased from 2.4% in 1992 to 13.9% in 2007 [9–11]. In 2007, only 0.7% of patients in US emergency departments used magnetic resonance imaging (MRI), underscoring the importance of CT availability.

CTs have increased their diagnostic capability while reducing examination times and radiation doses. At present, the state-of-the-art equipment performs real-time image acquisition with 320 “slices” (cross-sections used for reconstructing an anatomical image) or 640 slices interpolated, allowing for detailed visualization of moving organs. The 16-slice and higher models are recommended for angiographic exams, while more than 64 slices are needed for cardiologic studies. However, the introduction of more modern and expensive technology does not contribute to the cheapening of a simpler equipment, leading, instead, to its removal from the market. Additionally, the acquisition of more modern CTs frequently aims at productivity gains [12]. However, while technological advancements increase image quality, this increase may not be cost effective. For instance, some single-slice CTs can analyze 0.8 mm anatomical structures, a degree of detail sufficient for many clinical indications in daily practice [13].

This study aimed to compare models, evaluate their productivity, and identify the simplest (entry-level) model CTs offered in the world market, focusing on developing countries. Taking the results into account, a way to disseminate this equipment is then suggested.

The approach of the present work is that the replacement of simple CT models with more efficient and “sophisticated” ones neither meets the market requirements nor adequately serves the need of people in developing countries. Therefore, this information should be available for medical device companies hoping that they take this into account when making their market analyses and strategies.

## 2. Materials and Methods

### 2.1. Studied Devices

In this study, “productivity” was defined as the number of CT exams per unit of time. To provide a broad overview of the equipment and considering that even single-slice models are still used in many countries [11], models with one, two, four, 16, and 128 slices were included in the study.

For time measurements, we considered exams conducted using nine devices, from brands A (1-, 4-, and 16-slice) and B (2- and 128-slice), in eight private health facilities located in four cities of the state of Rio de Janeiro, Brazil (three specialized radiology clinics, one general medicine clinic, and four general hospitals). Four single-slice CTs were studied. One was an older model (1998), and the other three represented more modern models from 2003 (one CT) to 2009 (two CTs). The dual-slice model was manufactured in 2008, the 4-slice model in 2005, the two 16-slice models in 2011, and the 128-slice model in 2014. Given the differences mentioned in their manufacturing years, the single-slice scanners' processing times were analyzed separately.

### 2.2. Measurements

Exam procedures were divided into (1) the arrival of patient records to the examination room, (2) patient arrival, (3) patient positioning, (4) data input prior to exam, (5) image acquisition, and (6) patient departure.

Time was measured by two researchers using a stopwatch application with a precision of one hundredth of a second. Values were rounded, resulting in measured times in minutes (′) and seconds (″). The average times for and standard deviations of each exam step were calculated from the measured times.

Considering 66 working hours/week (shifts of 12 hours from Monday to Friday and 6 hours on Saturdays, typical for the studied units) and a constant distribution of exams, productivity was calculated by dividing the weekly working time by the total exam time for each model.

### 2.3. Evaluation of the International CT Supply

After obtaining the measured times, an internet search was performed to identify full-body CT manufacturers. Once a manufacturer's site was located, a more detailed search took place, looking for their market entry-level models (the simpler options for each geographical market) and trying to understand the availability of easily accessible devices to poorer countries. This search took place between February and December 2016.

## 3. Results

### 3.1. Exam Time

Table 1 presents the averages and standard deviations of the measured exam times, the percentage contribution of each stage, and the maximum weekly productivities. Times between record and patient arrival could not be obtained for the 128-slice model. The number of measured exam times was 15 for the single-slice models, six for the dual-slice models, 70 for the 4-slice models, 13 for the 16-slice models, and seven for the 128-slice model. Except for data acquisition, times were similar for the other stages in the different studied devices. The maximum estimated productivity of the 128-slice model was less than 2.5 times that of the simplest model (1998 single-slice).

### 3.2. CT World Market

The survey identified seven CT manufacturers. An eighth manufacturer produces a stationary model that was not included as it currently has a very small market share. Four manufacturers dominate the Brazilian market for CTs, but the other three also operate in Brazil, with a smaller presence.

Table 2 summarizes the offerings of CT entry-level models according to geographical region and manufacturer. Except for D and F (in Africa), all manufactures offer dual- or 4-slice entry-level models for Asia, South America, and Africa, and only C still offers single-slice entry-level models (in Asia). In the USA, models below 16-slice are offered by E and B [14–20].

## 4. Discussion

The aim of this study was to compare the productivity of CT models and to characterize CT entry-level offerings around the world. Medical equipment productivity is important for both private and public health services, in which waiting lists sometimes extend for months. However, the idea that doubling the number of CT slices per unit of time could double the number of exams (a linear increase) is not realistic, given that exam times are not exclusively dependent on the time needed for image acquisition/reconstruction [21].

This study indicates that the relationship between slices/rotation and productivity is not linear and that one cannot proportionally increase CT productivity by using models with “more slices.” For instance, exchanging a single-slice for a 16-slice model increased productivity by only a factor of 1.47. This observation draws attention to the importance of characterizing CT offerings around the world, given that, under these circumstances, many simpler models would still be adequate for regions or countries with less stringent demands and given that this offer is not well characterized, yet.

### 4.1. CT Exam Times

The duration of exam stages is similar, except for image acquisition times. Stages one, two, and six refer to the arrival and departure of a patient, while stages three, four, and five depend more directly on the device technology (ease of patient positioning, data input procedures, and speed of image acquisition). As expected, the 128-slice model was the fastest in terms of image acquisition and reconstruction. This equipment is designed for procedures that demand a large degree of detail but would be over-dimensioned for simpler exams.

However, times for remaining stages were similar in other models, and therefore, the differences between the average exam times were not large. For example, the average total time for exams in the 128-slice model was 42% less than that of the simplest analyzed model. These results quantify the fact that, without changes in exam protocols, even a very fast device would ultimately yield a small productivity gain relative to older models. For instance, for the 128-slice model, gains could be achieved by protocol changes in patient positioning and departure (the longest stages).

### 4.2. The World Market for Entry-Level CTs

In many countries, 1- to 4-slice CT models are still in widespread use. For instance, in Brazil, a 2014 study of 56 CTs in the State of Rio de Janeiro identified that 21% of these were dual-slice and another 21% were single-slice. [6] In this case, most entry-level models were between 2- and 16-slice, and single-slice models were available exclusively in Asia [14–20].

In 2013, in Brazil, a single-slice CT scanner costs approximately US$150,000; a dual-slice scanner, US$190,000; a 4-slice scanner, US$230,000; and a 16-slice scanner, US$320,000. However, as mentioned, for most examinations, even single-slice models may be adequate [13], and the productivity necessary for small towns can also be achieved with simpler models. Besides the cost problem, more sophisticated CTs usually have more complex and expensive maintenance, which becomes a more grave issue when one considers units located far from major urban centers. For example, a 2011 study identified that 85% of the 52 African hospitals studied had problems finding qualified local professionals to repair these devices [22]. Most African countries lack manufacturer offices, and therefore, their demands have to be met by offices in Europe or by local untrained personnel [23–25]. In Tunisia, a country with more than 10 million inhabitants, in 2011, there were 118 CTs and 21 MRIs, 70% of which are in private units [22].

### 4.3. A Low-Cost Solution: Shareable CT Platforms

From an engineering point of view, differences between slice numbers in CTs can be summarized as the following: (a) detectors—“multislice” models feature rows of detectors that determine the number of simultaneous slices (in the “single-slice” model there is only one row); (b) acquisition—more slices require greater capacity for data integration and more electronic boards; (c) collimation (defines the slice thickness)—“fan beams” in single-slice CTs are replaced by “cone beams” in multislice models; (d) communication—with increased data flow, transmission speed must increase with the number of slices; (e) design—the chassis (gantry and console) in the multislice models are larger than those in the single-slice models, as they house more parts; and (f) storage and image reconstruction—increasing the number of slices increases data processing needs, thus requiring more computing power [13].

A suggestion to enable the production of cheaper CTs would be to use “shareable platforms.” This practice is common in the automotive industry, with platforms used by many car models allowing interchange of components and generating an economy of scale. These platforms would share CT parts such as body, motors, cabling, couch, console, high voltage generator, X-ray tube, couch/gantry control boards, and gantry rotational/stationary communication systems. These kinds of CT could be included in programs such the WHO's WHIS-RAD that developed simple X-ray systems focused on developing country's needs [26].

In this way, a platform capable of holding up to a 32-slice system, for example, could receive a detector with only one row of detectors, collimators, boards, software, and hard disks (performing as a single-slice system). However, when necessary, the owner could request the addition/replacement of the necessary parts for an upgrade to a 4- to 32-slice system. These changes would not require the replacement of large parts such as the gantry or the couch, nor a new physical installation, and this approach would greatly reduce transportation and labor costs and equipment downtime. Thus, cheaper equipment would be made available for markets that lack more resources. Currently, there are already upgradable equipment (such as 16- to 32-slice), but there are no options for scaling up from simpler models. From a marketing point of view, providing such scalability would have the advantage of generating brand loyalty.

## 5. Conclusion

Differences among average exam times were not large, mainly due to exam stages not being directly related to data acquisition and image reconstruction. The increase in productivity due to the number of CT slices is not linear, and without organizational changes, a large productivity increase cannot be expected from changes to “faster” models. Therefore, CT equipment acquisition should consider variables such as the number and types of exams to be performed, keeping in mind that, in this context, “more” is not necessarily “better.” Especially for developing countries, opting for faster models may result in maintenance problems or in underutilization, justifying a more widespread offering of simpler models. The use of “modular” or “upgradeable” devices could create a new market, keeping entry-level devices economically competitive and increasing CT scan accessibility worldwide. This kind of strategy could be suggested and encouraged by IAEA, PAHO, and WHO as well as other international bodies such as the International Society of Radiology (ISR) and the International Organization of Medical Physics (IOMP).

## Figures and Tables

**Table 1:** Average and standard deviation (sd) of measured times in CT exam stages, stage percentage contribution (pc), total exam times, and theoretical maximum weekly exam productivities. Times in minutes (′) and seconds (^″^).

Exam stage		Single-slice (all)	Single-slice (modern)	Dual-slice	4-slice	16-slice	128-slice
Arrival	Average	39^″^	34^″^	2′02^″^	1′25^″^	40^″^	—
	sd	35^″^	14^″^	49^″^	2′43^″^	23^″^	
	pc	5.00	5.55	19.66	14.26	7.31	

Preparation	Average	2′09^″^	1′45^″^	1′30^″^	1′21^″^	32^″^	53^″^
	sd	1′45^″^	1′04^″^	48^″^	1′24^″^	31^″^	59^″^
	pc	16.50	17.13	7.30	13.11	5.85	15.92

Positioning	Average	1′23^″^	1′16^″^	1′50^″^	1′20^″^	2′33^″^	1′38^″^
	sd	35^″^	38^″^	38^″^	1′43^″^	1′49^″^	1′34^″^
	pc	10.64	12.40	16.85	13.11	27.97	29.43

Data entry	Average	1′09^″^	1′08^″^	1′13^″^	1′34^″^	56^″^	40^″^
	sd	41^″^	44^″^	30^″^	1′13^″^	22^″^	19^″^
	pc	8.85	11.09	11.66	15.25	10.24	12.01

Image acquisition	Average	5′38^″^	3′27^″^	2′05^″^	2′31^″^	2′41^″^	44^″^
	sd	4′35^″^	1′30^″^	31^″^	1′24^″^	1′11^″^	32^″^
	pc	43.33	33.77	17.56	24.75	29.43	13.21

Patient departure	Average	2′02^″^	2′03^″^	3′12^″^	1′59^″^	1′45^″^	1′38^″^
	sd	58^″^	1′05^″^	1′36^″^	1′24^″^	1′14^″^	25^″^
	pc	15.64	20.07	26.97	19.51	19.20	29.43

Total	Average	13′00^″^	10′13^″^	11′52^″^	10′10^″^	9′07^″^	5′33^″^
	sd	5′34^″^	2′52^″^	2′56^″^	5′11^″^	4′08^″^	4′45^″^

Productivity		304	387	333	389	434	713

**Table 2:** CT entry-level model (by the number of slices) offerings, by manufacturer and geographical region. “Europe” means that the site redirects to Europe. Internet search concluded in December 2016.

Region	A	B	C	D	E	F	G
USA/Canada/Mexico	16	6/6/2	40/16/2	16	2	16	
Central America			2			16	
Brazil/South America	4	2	2	16		16/2	2
Africa	4	2	Europe			16	
Oceania	16	2	16			16	16
Asia	4	2	1	16	2	2	
Europe	16	2	2	16		16	
Global (single site)					2		
